# Natural processes influencing pollinator health

**DOI:** 10.1098/rstb.2021.0154

**Published:** 2022-06-20

**Authors:** Philip C. Stevenson, Hauke Koch, Susan W. Nicolson, Mark J. F. Brown

**Affiliations:** ^1^ Royal Botanic Gardens, Kew, Kew Green, Richmond, Surrey TW9 3AE, UK; ^2^ Natural Resources Institute, University of Greenwich, Kent ME4 4TB, UK; ^3^ Department of Zoology & Entomology, University of Pretoria, Pretoria 0002, South Africa; ^4^ Royal Holloway University of London, Egham, Surrey TW20 0EX, UK

**Keywords:** pollinator health, nectar and pollen chemistry, bee microbiome, pollinator community, pollen nutrition, floral landscape

## Abstract

Evidence from the last few decades indicates that pollinator abundance and diversity are at risk, with many species in decline. Anthropogenic impacts have been the focus of much recent work on the causes of these declines. However, natural processes, from plant chemistry, nutrition and microbial associations to landscape and habitat change, can also profoundly influence pollinator health. Here, we argue that these natural processes require greater attention and may even provide solutions to the deteriorating outlook for pollinators. Existing studies also focus on the decline of individuals and colonies and only occasionally at population levels. In the light of this we redefine pollinator health and argue that a top-down approach is required focusing at the ecological level of communities. We use examples from the primary research, opinion and review articles published in this special issue to illustrate how natural processes influence pollinator health, from community to individuals, and highlight where some of these processes could mitigate the challenges of anthropogenic and natural drivers of change.

This article is part of the theme issue ‘Natural processes influencing pollinator health: from chemistry to landscapes’.

## 1. Introduction

Animal pollination is one of nature's most compelling mutualisms: plants offer a reward to floral visitors in exchange for the transfer of pollen between flowers to facilitate plant reproduction. Pollination services support a major component of global food production but are also critical to natural ecosystems [1,2]. However, evidence from recent decades indicates that pollinator abundance and diversity are at risk, with many species in decline [3–6].

Research identifying the causes of pollinator decline has focused on anthropogenic drivers, including pesticides, habitat loss and climate change and interactions of these constraints [7–10]. That these constraints have detrimental impacts on pollinators is broadly understood and accepted. However, the natural processes that influence pollinator health and may contribute to or even mitigate declines are, by comparison, overlooked. Understanding these processes is vital for the development of nature-based solutions that support healthy pollinators and restore their diversity and abundance. For example, pollen and nectar chemistry and the pollinator microbiome can influence pollinator health [11–15]. Furthermore, landscapes are increasingly described with respect to their specific nutritional value to pollinators rather than simply floral diversity or abundance [16,17]. Here we redefine pollinator health from a community perspective, critically assess some of the natural processes that influence pollinator health, and identify natural drivers of change and potential nature-based solutions to the existential challenges facing pollinators.

Historically, pollinator health has referred simply to honeybees and specifically honeybee diseases and parasites. As the importance of wild bees and other pollinators to food production and natural habitats has become better understood [18], there is increasing reason for pollinator health to include all pollinators at different ecological levels. Pollinator health must also be understood with respect to a multitude of drivers and how they influence the full spectrum of species.

## 2. Natural processes influencing pollinator health: from the top down

Pollinator health has traditionally been approached by focusing on the individual, or by using a hierarchical and reductionist approach, working from internal processes through to the health of the population or species. For example, López-Uribe *et al*. [19, p. 271] focused on honeybees, and defined health as ‘the state of well-being that translates into the ability of organisms to acquire, allocate and use energy optimally to increase fitness'. De Miranda *et al.* [20] took this further by applying a One Health perspective to a range of pollinating bees, generating a practical working definition of bee health, which enabled them to identify a set of potential metrics for identifying bee health in the field. In parallel, Parreño *et al*. [17] have recognized that pollinator health is influenced by multiple biological processes and environmental factors, and highlight the importance of nutritional niche space to pollinator health in the context of wild species of bees. However, such hierarchical reductionist approaches may miss key traits of pollinator health at the community level.

**Figure 1.**
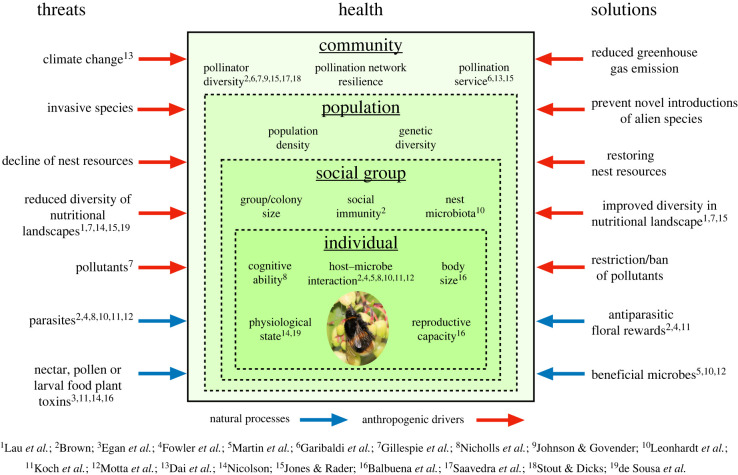
Natural processes and anthropogenic drivers influencing pollinator health and potential mitigating solutions at community, population, social group and individual levels expanded from López-Uribe *et al.* [19]. (Online version in colour.)

Here we expand on López-Uribe *et al.* [19] and propose an ecosystems-level approach, starting at the level of the pollinator community and its provision of pollination services (figure 1). From this perspective, pollinator health can be argued to be analogous to the stability, robustness or resilience of the pollinator community to environmental change. Network metrics can be used to assess the health of a community [21], as can simple measures of abundance, richness and diversity. Arguably, a trait-based approach, where similar species can be considered as functional replacements, might be useful in this perspective. Thus, health at the community level might not be impacted by the loss or reduction of one species (ill-health) if it is naturally replaced by a functionally similar species. Concomitant with this, factors such as pathogens that, at the level of individuals, might be considered as detrimental to health, could play important positive roles at the community level in maintaining species diversity, and thus community health [22]. Consequently, factors that have been previously viewed solely through the lens of pollinator health at the individual or population level, such as food availability, food quality, parasites, pathogens, and secondary chemicals that enable medication, need to be reconsidered at the pollinator community level. A definition of pollinator health at this level might mean that a healthy pollinator community is resilient in the face of environmental perturbations and provides a robust pollination service.

Of course, such an ecosystem-led view does not mean that we can simply ignore the impact of environmental factors on the health of individual pollinators. Robustness and resilience at the ecosystem level need to be supported by health at the individual level, even if that does not mean equal health for every individual within every species, and so understanding how factors such as nutritional quality drive individual health, and, ultimately, reproduction, remains key. For pollinator communities to be stable in the long term, their individual components need to be healthy enough to reproduce and contribute to the next generation. Indeed, most papers in this special issue examine health at the level of individuals, with only a few focusing on the community level. We believe that incorporating a community definition of pollinator health that integrates health at the level of individuals, colonies, and populations within communities provides the path toward maintaining wild pollinator communities and the critical services they provide into the future.

## 3. Floral chemistry influences on pollinator health and behaviour

Secondary metabolites have been reported frequently in nectar and pollen [23–26], although there are surprisingly few examples reporting their effects on pollinator behaviour and health [13,24]. This may reflect challenges historically in instrumentation and analysis of compounds only available from very small sample sizes in low concentrations. Modern and highly sensitive instruments such as liquid chromatography–mass spectroscopy (LC-MS) have opened up this field.

Most compounds occurring in nectar and pollen are also recorded elsewhere in the plant [27], where many also provide a defensive function against antagonists; thus their presence in the floral reward for pollinators is a paradox [28]. For example, the insecticidal diterpene grayantoxin 1 is a defence compound against thrips in the foliage of *Rhododendron simsii* [29]. The same compound occurs in *Rhododendron* nectar at concentrations that are toxic to honeybees and mining bees whereas, conversely, bumblebees are unaffected [30]. This differential toxicity alludes to a chemical-based specialist pollinator syndrome. If consumed, these compounds could present a health challenge to bees at individual and colony levels, but for bumblebees the flowers may provide a surfeit of food since few other flower visitors can tolerate the toxins. In Ireland, the number of *Bombus pascuorum* nests in the vicinity of *R. ponticum* is almost double the number recorded elsewhere [31]. Whether this presents an adaptation by the plant to optimize pollination service or adaptation by bees to the toxin is not clear. However, invasive populations of *R. ponticum* in the British Isles show reduced toxin levels, suggesting that plants have modified their chemistry in response to an otherwise poorly adapted pollinator community [32]. Honeybees avoid grayanotoxin given a choice, so it does not present an individual or higher ecological tier health risk unless there are no alternative food sources. However, Egan *et al*. [33] report that pollinators impose negative directional selection against grayanotoxin in nectar of invasive *R. ponticum*, which contrasts with selection patterns quantified in the species' native range, where this compound was under positive selection in nectar. Nectar concentrations were decoupled from those of leaves in the invasive but not the native range, which is likely to assist this species to evolve and facilitate visits by pollinators while simultaneously maintaining anti-herbivore defence.

Secondary metabolites may therefore have multiple functions for plants and drive interactions with mutualists and antagonists. This has been illustrated with caffeine, a widely distributed plant alkaloid that reportedly provides a defensive mechanism against insects through toxicity or feeding inhibition [34–36], behaviour-modifying effects at individual [37,38] and colony levels [39], as well as anti-parasite activity against microsporidian parasites of bees (*Nosema* spp.) [40,41]. Indeed, the bioactivity of nectar compounds against bee pathogens illustrates the most direct pollinator health impact of floral chemistry at the individual level. Compounds reported to occur in nectar or honey were evaluated against the gut parasite *Crithidia bombi* and shown to have antimicrobial activity, suggesting potential to mitigate the challenge of excessive disease burden [12]. More recently, acquisition of *C. bombi* by *Bombus terrestris* was shown to be significantly reduced in bees feeding on the *Calluna vulgaris* (ling heather) nectar metabolite, callunene [11]. Since *B. terrestris* feeds on heather nectar naturally, and nectar from this species is the third most abundant in the UK [16], this provided the first example of an ecologically relevant and widely available disease-mitigating benefit to pollinator health. However, callunene was not recorded in the hindgut, where parasites are most abundant, suggesting it had been metabolized, and consequently that established infections were not affected when this compound was consumed by a *Crithidia*-infected bee. Koch *et al*. [42] provide a possible explanation for this through a study of the interaction of *B. terrestris* with nectar compounds from lime (linden) tree flowers (*Tilia* spp.) and strawberry trees (*Arbutus unedo*). Unedone from *A. unedo* nectar was inhibitory to *C. bombi in vitro* and in *B. terrestris* gynes, whereas tiliaside in *Tilia* nectar was only inhibitory *in vivo*. This is because tiliaside was deglycosylated by the bumblebee during gut passage, increasing its antimicrobial activity in the hindgut, the site of *C. bombi* infections. Conversely, unedone was inactivated by glycosylation in the midgut by the bumblebee, only to be deglycosylated by the microbiome in the hindgut, restoring its activity. Koch *et al*. [42] thus demonstrate that metabolism of nectar compounds by the host or the microbiome modifies their antiparasitic activity.

When pollinators use floral resources but their larval stages feed on the foliage of the same plant, there is an ecological conflict and a challenge for the plant to mediate these interactions. Balbuena *et al*. [43] present one such example in *Hyles lineata,* a common hawkmoth that feeds on the flowers of *Oenothera harringtonii* whereas the larvae feed on its leaves. They monitored growth, survival and fecundity as individual-level measures of pollinator health and showed that the plant modifies floral and foliar chemistry to optimize the services of pollinators while protecting against herbivory using a complex of constitutive and induced chemical processes. The larvae of *H. lineata,* however, perform well on other related species of *Oenothera,* suggesting that in asymmetric plant–pollinator interactions alternative larval host plants are critical in maintaining pollinator health.

Mammal pollination systems have evolved in several plant families, and while some research has identified drivers of interactions between flowers and bat pollinators [44,45], there are substantial gaps in our knowledge. One outstanding question is whether sensory bias evolved to facilitate intraspecific communication or for seeking food. There are several examples of ground-dwelling mammal pollination systems in southern Africa, many of the pollinators being nocturnal and so reliant on scent. The quantities of nectar produced by the host species for mammalian pollinators are typically far greater than those provided by insect-pollinated species, so adapted to suit a specific dietary requirement. These plants flower in winter when other food for rodents is scarce. To ensure the mammalian pollinator is healthy and able to continue to provide pollination services, the floral cues provided by the flowers to attract the pollinator are critical in enabling these pollinators to find the right food. Johnson & Govender [46] report that four species of rodents were broadly attracted to oxygenated aliphatic nectar chemicals such as esters and ketones but not to aromatics (conjugated planar rings such as benzyls) which occur frequently in the floral odour of insect-pollinated plants, nor to a sulfide compound that is attractive to bats. The attractiveness of some of the ketones and esters was lost when combined with unattractive compounds, suggesting the overall chemical environment is important. These volatile floral chemicals facilitate the exploitation of rodent sensory bias that likely evolved in intraspecific communication or searching for seeds.

## 4. Nutrients in nectar and pollen and their importance for pollinator health

Poor nutrition results from the loss of natural habitat and from extensive monoculture plantings, and diminishing forage is understood to be a major cause of pollinator declines [7–9]. Good nutrition, however, can offset stresses from pesticides and diseases. Overall, diverse and continuously available forage leads to more balanced nutrition and access to beneficial phytochemicals.

Nectar is an energy source for most pollinators. Nicolson [47] provides a broad synthesis of nectar chemistry and nutritional quality, including implications for vertebrate pollinators as well as bees. The historical context of research on nectar chemistry is touched on, but also recent metabolomic studies (e.g. [48]). A model of the mechanisms of nectar secretion [49] offers a simple explanation for the differences in nectar volume and sugar composition which have stimulated much research on the association between sucrose proportion in nectar and pollinator type. These patterns are particularly clear for nectar-feeding birds and their flowers. Apart from direct nutritional benefits, many nectar compounds such as amino acids and secondary compounds have indirect effects on foraging behaviour and parasite infection. Water, usually ignored in the composition of nectar, is also a nutrient, and the water component of nectar is a major factor in its variability but also important for consumers. Phenotypic variation in nectar chemistry is common [50], and there is increasing evidence for effects of microbial contamination on nectar chemistry [51].

Pollen is more difficult to analyse. It varies widely in nutrient composition [52,53], but much of this variation may be due to discrepancies between the methods used in pollen analysis. Differences in methods make it difficult to compare studies. In this issue, Lau *et al*. [54] review the common methods used to analyse pollen protein and lipids—the macronutrients most often linked to bee health. Using *Brassica* and *Rosa* pollens, they compared a subset of these methods while also carrying out a more complete analysis. Pollen has unique physical properties and it is demonstrated here that fracturing pollen grains can lead to marked increases in estimates of protein and lipid content. Fracturing may be particularly necessary for complete extraction of components such as fatty acids, which are critical for pollinator fitness [55]. Fortunately, the widely used Dumas combustion assay for nitrogen (protein) does not require this. The authors recommend the use of standardized methods to facilitate comparisons between independent studies. In addition, disrupting pollen grains before analysis, while more important for some pollens than others, may greatly reduce the variation in data on nutrient content.

The analysis of *Brassica* and *Rosa* pollens [54] included major elements: this area of pollinator nutrition is receiving increased attention and may be important for the health of honeybee colonies [56]. De Sousa *et al.* [57] tested the dose-related responses of young worker honeybees in cages to mineral-laced sucrose solutions. They selected the minerals most prevalent in pollen, the major source of micronutrients for bees; it is easier to study responses to minerals in solution. They divide the eight minerals tested into salts and metals: however, all are metal ions that play essential roles in insect physiology, especially transport processes and enzymatic activity [58]. Honeybees showed some regulatory ability and avoided high and potentially toxic concentrations of all minerals used except Na: this is in agreement with Bertrand's rule, which predicts that low concentrations of micronutrients will be attractive and high concentrations will be repellent. Honeybees also obtain minerals from nectar and water [59]. Sodium is scarce in the diets of herbivores, and enriching floral nectar with sodium attracts more pollinator visits and more species [60].

The larval diets of solitary bees are a mixture of pollen and nectar with added microbes. Leonhardt *et al.* [61] investigated the amino acid and fatty acid profiles of pollen provisions in the solitary megachilid bee *Osmia bicornis*, and whether these nutrients are correlated with bacterial microbiomes in the bees and their provisions. Bee larvae and pupae and larval provisions were sampled from different populations using trap nests at sites differing in land use and thus floral resources. Pollen types in provisions were identified and the nutrients analysed. Bacterial communities of pollen provisions and bee guts showed strong overlap. Pollen-derived bacteria may play an important role in amino acid and fatty acid provisioning; on the other hand, amino acids and fatty acids in the pollen provisions may favour particular microbial communities. The authors use neural network analysis to show correlations between amino and fatty acids and bacterial genera, but it is not possible to say whether specific nutrients were synthesized by plants or bacteria (or both). Microbial interactions may explain why larvae of both specialist and generalist bees often fail to develop on unsuitable pollen diets [62].

The final paper in this section looks beyond bees to include other insect pollinator taxa and addresses pollination at the landscape scale. Jones & Rader [63] broadly review the nutritional challenges for pollinators in agroecosystems, emphasizing the need to maximize not only bee diversity and abundance but also crop pollination outcomes. Preserving remnant habitat and introducing extra floral resources do not necessarily improve pollinator health or crop yields. The challenge is that much more information is needed on the nutritional needs of specific pollinator taxa and the resources that provide them. Even for bees, most of the available information on nutritional ecology is for a limited number of species: *Apis mellifera*, *Bombus* and mason bees (*Osmia*) [64]. Traditional and new approaches to evaluating nutritional requirements are outlined here and by [65]. Some of these methods can be applied to non-bee taxa. There is also a compelling need to redress the geographical bias in crop pollination studies [66].

## 5. Microbial influence on pollinator health

Microorganisms are major drivers of pollinator health. On the scale of the individual, effects of microbial associates on host health form a continuum from the negative impacts of parasites to benefits derived from symbionts, and can change on ecological or evolutionary time scales [67].

While parasites of pollinators can reduce individual health parameters such as reproductive capacity, foraging ability and physiological state, hosts can reduce negative effects of parasites through the action of their immune system, or through specific diets with medical antiparasitic effects. A better understanding of the natural mechanisms by which pollinators are able to prevent, reduce or tolerate parasite infections may inform pollinator conservation decisions, if they are, for example, linked to the availability of certain nectar or pollen sources in the environment [68]. Certain diets can reduce parasite infections in pollinators, for example through the antiparasitic activity of nectar secondary metabolites ([42]; see discussion above). Direct chemical effects of specific diets on parasites may, however, not be the only mechanisms of antiparasitic action. A sunflower pollen diet has, recently, been shown to induce strong and consistent reduction in the infections of bumblebees with the gut parasite *C. bombi* [69], but, so far, chemical constituents of sunflower pollen have not been shown to induce this effect [70]. In this special issue, Fowler *et al*. [71] test if the antiparasitic effect of sunflower pollen could instead derive from a modulation of the immune response of bumblebees. Bumblebees feeding on a sunflower or wildflower control diet did not differ in their induced or constitutive immune responses as measured by the activity of phenoloxidase and the humoral antibacterial activity of haemolymph. This suggests that the antiparasitic effects of a sunflower pollen diet are either linked to immune parameters (although these were not measured), or derive from a different, as yet unknown mechanism.

Beneficial microbial symbionts of pollinators can improve pollinator health through digesting or detoxifying diet components, defending against parasites, or stimulating immune and metabolic pathways of the host. Motta *et al*. [72] review the existing literature on these health benefits derived from the bacterial microbiome of social corbiculate bees (honeybees, bumblebees, stingless bees), and present new data on the potential of inoculating honeybees with probiotic bacteria as a way to improve their health. They highlight that stressors like antibiotics or poor diet may disrupt the bee microbiome, and lead to increased disease susceptibility. Administering probiotic bacteria to bees has the potential to restore health-promoting microbiomes, but experimental evidence for the promise of this approach is largely missing. Motta *et al*. [72] experimentally show that commercially available probiotics with bacteria that are not natively found in the honeybee gut fail to colonize honeybees, while cultured native bacterial strain colonies efficiently and induce the activation of immune and metabolism genes. This suggests existing probiotics may have limited or no benefits for honeybees, but future probiotic research in bees should focus on using bacterial strains with beneficial health effects naturally found in bees.

Martin *et al*. [51] looked beyond the endogenous gut microorganisms of pollinators, and review the potential effects of nectar microbes on pollinator health. Bacteria and yeasts in nectar alter its chemical composition, with negative (e.g. reduced sugar content) or positive (e.g. increased amino acid content, increased amounts of micronutrients like vitamins and sterols) effects for pollinator nutrition and health. Pollinators may modulate their foraging behaviour based on microbial presence in nectar, likely through detecting volatile organic compounds released by nectar microbes. This may facilitate the detection of nectar sources for pollinators and may affect pollination services on a landscape scale. Martin *et al*. [51] also argue for more research into the effects of nectar microbes on disease dynamics in pollinators, as these microorganisms could affect floral transmission of pollinator pathogens, or infections within pollinators, for example through the production of antibiotic compounds by floral yeasts.

Nicholls *et al*. [73] highlight the importance of foraging behaviour for disease dynamics of pollinators. Horizontal transmission of pollinator pathogens often occurs on flowers [74,75]. A better understanding of the factors affecting floral pollinator disease transmission, such as floral traits and effects of flowering plant species diversity, may inform a better design of managed landscapes to reduce the spread of pollinator diseases. Existing studies in part show contradictory patterns for this interaction [73], but investigating effects of different foraging behaviour of diverse pollinator species on disease transmission may help resolve this.

Brown [22] provides an important community- and landscape-level view of pollinator health, which argues for considering pollinator parasites as an integral part of biodiversity. While most research on pollinator health has focused on the detrimental effects of parasites on individual or colony host health, at a landscape level, parasites may facilitate coexistence of diverse pollinator communities, and are major natural drivers of evolutionary dynamics. Therefore, Brown [22] argues that natural host–parasite interaction networks should be conserved, rather than eliminated. A better understanding of the impacts of floral rewards on host–parasite interactions may be used to design landscapes that support pollinators to moderate levels of parasite infections and ensure pollination services.

## 6. Landscape, society and pollinator health

The landscapes in which pollinators exist in the so-called Anthropocene are ultimately determined, and increasingly so, by human actions. Consequently, the long-term maintenance of healthy pollinator communities relies upon positive, evidence-based and informed actions across all levels of society, from individuals to global bodies. However, a key difficulty we face in recommending such action is our limited understanding of causal drivers of pollinator health in natural and semi-natural systems. Given the range and variety of these drivers, many of which are covered in this issue, the experimental work to investigate this is simply too great [76]. By contrast, Saavedra *et al.* [77] provide a statistical approach that could enable us to understand causal drivers of, for example, pollinator richness, based on observational rather than experimental data. Given the wealth of observational data in the scientific literature, and the relative ease with which it can be collected (as opposed to the cost and complexity of ecological experiments), application of probabilistic systems analysis rooted in nonparametric causal inference holds out real hope for the scientific community to take apart the complex relationships between pollinator communities and ecological and environmental drivers.

Once recommendations have been identified, based either on sound experimental or statistical evidence, how can we implement them most effectively? Numerous pollinator conservation initiatives have been put in place around the globe, but are they on a sound footing? And how can they be improved? Stout & Dicks [78] analyse current initiatives and present an analysis of the key elements that are needed for effective societal interventions to support pollinator health. Crucially, they also identify higher-level issues—such as patterns in global trade—that need to be addressed if we are to support pollinator health, and arguably ecosystem health and biodiversity more broadly. Future pollinator conservation initiatives designed to incorporate the results of this analysis would be significantly enhanced.

The direct link between pollinator health and human health could help highlight the importance of healthy pollinator communities and pollination services. In this issue Garibaldi *et al*. [79] show that very few studies have evaluated aspects of pollinator health and human health together, and these contributions were limited to the fields of nutrition, medicine provisioning, mental health and environmental quality. Benefits are provided through more nutritious food, an estimated 28 000 animal-pollinated medicinal plants, products such as honey, the maintenance of green landscapes that enhance mental well-being, and sustainable practices associated with pollinators. This suggests that pollinator diversity could be a proxy for the benefits that landscapes provide to human health.

While human impacts of pesticides and climate change on pollinators have received much attention for their direct impacts on pollinators [3], other anthropogenic activities could indirectly influence natural processes with consequences for pollinator health. Climate change, for example, affects the distribution and phenology of pollinators and plants, and leads to changes in floral rewards associated with temperature and water availability. Dai *et al.* [80] carried out a long-term study of soil moisture effects on *Gentiana aristea* in an alpine region on the Tibetan Plateau, and found that water stress in either direction affected floral traits, pollinator attractiveness and seed production. These changes were linked to greater allocation of resources to roots and stems during water stress.

## 7. Conclusion

Healthy pollinators live longer and reproduce more, and therefore support pollination services more effectively, even in the presence of pathogens. While the focus of study has been anthropogenic drivers of change, here we argue that pollinator health is also influenced by a range of natural processes, including nutrient availability secondary metabolites, beneficial microbes, diseases and predators as well as habitat and landscape changes. Consequently, an understanding or analysis of pollinator health must consider these natural processes, especially when seeking to mitigate against constraints that have a negative influence on pollinators. Understanding pollinator health at multiple levels of vigour, resilience and function not only in the context of individuals, colonies and populations but at the community level is also essential to address the drivers of poor health from floral chemistry and nutrition through to landscapes to assess vulnerability, adaptability and the impact of different environments or stressors on different species. At the community level, pollinator health and resilience reflect sustained pollinator diversity over time, considering both richness and evenness of pollinator species. Adopting such a community-level perspective will transform ecosystem management for healthy and effective pollination services to crop production and natural landscapes.

## Data Availability

This article has no additional data.
